# Pediatric Challenges in Robot-Assisted Kidney Transplantation

**DOI:** 10.3389/fsurg.2021.649418

**Published:** 2021-03-25

**Authors:** Julien Grammens, Michal Yaela Schechter, Liesbeth Desender, Tom Claeys, Céline Sinatti, Johan VandeWalle, Frank Vermassen, Ann Raes, Caroline Vanpeteghem, Agnieszka Prytula, Mesrur Selçuk Silay, Alberto Breda, Karel Decaestecker, Anne-Françoise Spinoit

**Affiliations:** ^1^Department of Urology, ERN eUROGEN Accredited Centre, Ghent University Hospital, Ghent University, Ghent, Belgium; ^2^Department of Vascular and Thoracic Surgery, Ghent University Hospital, Ghent University, Ghent, Belgium; ^3^Department of Pediatric Nephrology and Rheumatology, ERN ERKNet Accredited Centre, Ghent University Hospital, Ghent University, Ghent, Belgium; ^4^Department of Anesthesiology and Perioperative Medicine, Ghent University Hospital, Ghent University, Ghent, Belgium; ^5^Division of Pediatric Urology, Department of Urology, Biruni University, Istanbul, Turkey; ^6^Department of Urology, Fundació Puigvert, Universidad Autonoma de Barcelona, Barcelona, Spain

**Keywords:** pediatric kidney transplantation, kidney transplantation, pediatric robot-assisted kidney transplantation, robot-assisted kidney transplantation, robotics, robotic surgery, minimal-invasive surgery

## Abstract

Kidney transplantation is universally recognized as the gold standard treatment in patients with End-stage Kidney Disease (ESKD, or according to the latest nomenclature, CKD stage 5). Robot-assisted kidney transplantation (RAKT) is gradually becoming preferred technique in adults, even if applied in very few centra, with potentially improved clinical outcomes compared with open kidney transplantation. To date, only very few RAKT procedures in children have been described. Kidney transplant recipient patients, being immunocompromised, might be at increased risk for perioperative surgical complications, which creates additional challenges in management. Applying techniques of minimally invasive surgery may contribute to the improvement of clinical outcomes for the pediatric transplant patients population and help mitigate the morbidity of KT. However, many challenges remain ahead. Minimally invasive surgery has been consistently shown to produce improved clinical outcomes as compared to open surgery equivalents. Robot-assisted laparoscopic surgery (RALS) has been able to overcome many restrictions of classical laparoscopy, particularly in complex and demanding surgical procedures. Despite the presence of these improvements, many challenges lie ahead in the surgical and technical–material realms, in addition to anesthetic and economic considerations. RALS in children poses additional challenges to both the surgical and anesthesiology team, due to specific characteristics such as a small abdominal cavity and a reduced circulating blood volume. Cost-effectiveness, esthetic and functional wound outcomes, minimal age and weight to undergo RALS and effect of RAKT on graft function are discussed. Although data on RAKT in children is scarce, it is a safe and feasible procedure and results in excellent graft function. It should only be performed by a RAKT team experienced in both RALS and transplantation surgery, fully supported by a pediatric nephrology and anesthesiology team. Further research is necessary to better determine the value of the robotic approach as compared to the laparoscopic and open approach. Cost-effectiveness will remain an important subject of debate and is in need of further evaluation as well.

## Background

Kidney transplantation (KT) is universally recognized as the gold standard treatment in the adult and pediatric populations with patients with End-Stage Kidney Disease (ESKD).

In contrast to the adult population, KT has taken longer to become an established and preferred treatment for pediatric patients with ESKD (1). KT offers major advantages to the pediatric population such as catch-up growth (2) and longer survival (3). Immunosuppresive treatment increases the risk for perioperative surgical complications which creates additional challenges in management of kidney transplant recipients. Specifically, wound-related complications have been shown to contribute significantly to morbidity post-transplant (4). Applying techniques of minimally invasive surgery may contribute to the improvement of clinical outcomes for the pediatric ESKD population and help mitigate the morbidity of KT.

The past decade has produced greatly improved KT outcomes largely due to enhanced immunosuppressive therapy, expanded living kidney donor transplantation, advances in perioperative care, better pre-transplant preparation of the recipient and improved surgical techniques in kidney extraction from donors. At present, survival rates at 1-, 5- and 10-years post-transplant are 98, 95, and 91%, respectively (5). Survival rates are higher in recipients of living donor kidneys as compared to recipients of deceased donor kidneys (5). It should be noted that patient comfort is higher the procedure is pre-emptive, which is usually thanks to living donor, even if cadaveric donor is possible. Pre-emptive grafting means the patient does not have to undergo dialysis, the kidney graft is achieved before the patient is in need for dialysis.

## Robot-Assisted Laparoscopic Surgery

Progression in robot-assisted laparoscopic surgery (RALS) has revolutionized minimally invasive surgery. Although it is routinely utilized in urology, RALS has remained controversial since it is frequently challenging to demonstrate clear superiority in outcomes when compared to open or classical laparoscopic surgery, whilst also being quite expensive (6). Adaptation of RALS in children is even more hazardous than in adults as the medical equipment for the procedure is produced for use in adult-sized bodies (7).

Usage of RALS in the pediatric urology population was first described by Craig Peters's team in 2002 after they performed a pyeloplasty for an uretero-pelvic junction obstruction (Personal communication, Craig Peters, Children's Hospital in Boston, 2002). Since then, surgical indications for robotics in pediatric urology have expanded substantially. The principal robot-assisted (RA) procedures in children are RA pyeloplasty, RA hemi-nephrectomy, RA nephroureterectomy, RA ureteral reimplantation, RA bladder augmentation and RA Mitrafanoff appendicovesicostomy (8). Because of the insufficient evidence-base related to the lack of randomized-controlled trials, pyeloplasty surgery is the only procedure acknowledged by the European Association of Urology Guidelines on Pediatric Urology as equally successful whether performed using an open, laparoscopic or robotic approach (provided that the surgeon is experienced in the chosen technique) (9). Better cosmesis, less pain and less hospital stay are the main three advantages of robotic surgery stated in the guidelines.

Minimally invasive surgery such as laparoscopic surgery or RALS has consistently shown to produce improved clinical outcomes as compared to open surgery equivalents. These outcomes include fewer complications, reduced length of hospital stay, reduced need for blood transfusion, less postoperative pain, shorter convalescence period, fewer surgical site infections, and better cosmetic results (10–12).

RALS has been able to overcome many restrictions of classical laparoscopy, particularly in complex and demanding surgical procedures (e.g., kidney transplantation). Some major advantages include a superb three-dimensional vision, image magnification, elimination of hand tremor and movement scaling, control of the camera by the surgeon, good surgeon ergonomics to lessen fatigue, and articulated instruments with seven degrees of freedom of movement (13).

Despite the presence of these improvements, many challenges lie ahead in the surgical and technical–material realms, in addition to anesthetic and economic considerations (7, 8).

## Current Evidence

The first adult robot-assisted kidney transplantation (RAKT) was performed by Giulianotti et al. (14) in 2010. In 2014, Menon et al. (15) standardized the transperitoneal approach with regional hypothermia maintenance during the re-warming time. In the years since, RAKT has become an increasingly common procedure in selected high-volume centers. This can be attributed to promising results which have indicated RAKT to be a safe, feasible and reproducible procedure with a potentially improved morbidity as compared to open kidney transplantation (OKT) (16, 17).

However, this evidence is largely limited to adults (16, 18, 19). Children are not just ‘small adults' and must be assessed within their own separate population, established results in adults cannot be easily extrapolated to the pediatric population. To date, RAKT experience remains limited in the pediatric population.

The first full case report on pediatric (8 years old child) RAKT was published in 2019 by Decaestecker and team at Ghent University Hospital, Belgium. It demonstrated that RAKT in children is technically feasible and safe, and resulted in excellent graft function. Concomitant nephrectomy was done laparoscopically through the single-port GelPOINT® (AFS), while another team retrieved the living donor kidney using robotic assistance (KDK). In order to minimize ischemia in the donated kidney, the harvesting in the living donor and the transplant preparation of the child were simultaneously performed in two operating rooms by two different transplant teams (AFS—KDK). It should be noted that, given these demanding and complex aspects of the procedure, pediatric RAKT should only be attempted by an experienced RAKT team fully supported by a pediatric nephrology and anesthesiology team (20).

Recently, Bansal et al. released a comparative analysis of outcomes and long-term follow-up of pediatric RAKT with an open kidney transplantation (OKT) counterpart (21). Twenty-five patients were included in the study, 21 of whom underwent OKT, and four of whom underwent RAKT. A significantly higher re-warming ischemia time was noted in the RAKT group (21), but that may be attributable to the initial phase of the learning curve for the procedure, a phenomenon which was similarly observed in the initial phases of adult RAKT (16, 18). Peri-operative analgesic requirements were significantly higher among the OKT group as compared to the RAKT group. RAKT complications included transplant renal artery stenosis and subcapsular hematoma, albeit not exclusive to RAKT. Despite the limitations of this study, it demonstrated that pediatric RAKT is a feasible and safe procedure that results in excellent graft function (21).

In addition to these two articles, our literature search revealed two abstracts discussing pediatric RAKT. Modi et al. (22) described a group of 5 children undergoing RAKT in 2013. All but one kidney were issued from living donors. In two children, concomitant seminal vesicle cyst excision and nephrectomy were carried out. One child experienced spontaneous graft rupture on the 3rd postoperative day requiring emergency exploration (22). A prospective non-randomized open label trial by Patel et al. (23) compared outcomes of open vs. robot-assisted pediatric KT at a single center, involving 60 and 22 patients undergoing RAKT and OKT, respectively. Endpoints of their trial were feasibility of RAKT and creatinine value at 30 days post-transplantation. Both ureteric reimplantation time and intraoperative blood loss were significantly decreased in the RAKT group. In the RAKT group (*n* = 60), five patients had slow graft function, and seven patients lost graft during follow-up. Two patients had acute antibody-mediated rejection, four had chronic rejection, and one had de novo collapsing glomerulopathy with BKV nephropathy. Five patients from the RAKT group deceased during follow-up. In the OKT group (*n* = 20), two patients had slow graft function, two lost graft, and two deceased during follow-up. There was no significant observed difference in graft survival or overall survival (23). Both Modi et al. and Patel et al. (22, 23) concluded that RAKT is feasible and safe, however, a more thorough and comprehensive analysis of the specifics of their experiences could help increase the external validity of their findings.

## Our Experience

Based on our extensive experience in RALS in pediatric urology (24–26) and robotic kidney transplantation surgery (16, 27, 28), we decided to initiate a pediatric robot-assisted kidney transplantation program. For the time being, we have limited the program to cases of grafting performed with living donor transplants. Since the first pediatric RAKT case in 2018 (20), we have operated on an additional two children with good results, and our program has continued to advance. We continue to monitor our data and hope to further develop our experience in this unique field and improve patient outcomes.

## Discussion

End-stage Kidney disease (ESKD) occurs in about 5–10 children per million each year (29). Although early pediatric KT was complicated by technical, immunologic, and logistic problems, advances in these areas have led to dramatic improvements and KT has become the preferred treatment for pediatric ESKD patients (1). Innovations in minimally invasive surgical techniques that have been successful in the adult population should be offered to children as well if the benefits are comparable. Nonetheless, several considerations must first be addressed regarding different aspects of a RAKT.

## Anesthetic Considerations for RALS

Anesthetists should consider the following challenges in robotic surgical case: increased abdominal pressure, hypothermia, increased CO_2_ absorption, and the physiologic effects of Trendelenburg position. These may impair cardiovascular function and ventilation, induce cerebral vasodilatation, and negatively affect urine output (30). Anesthetists should take note that intra-abdominal pressures lower than 10 mmHg do not appear to induce any significant clinical hemodynamic effects, and pressures up to 12 mmHg seem to be well-tolerated (31).

## Surgical Challenges of RALS in Children

The relatively small size of the abdominal cavity of a child presents multiple operative challenges. This decreased workspace in pediatric abdominal surgeries makes trocar placement a critical procedural component as it can impact desufflation risk, bleeding risk, and organ damage. Poorly positioned trocars may generate a gas leak, provoking rapid desufflation and further decreasing the small workspace (32). The use of the AirSeal® system can help to maintain a stable working space with a very low pneumoperitoneum pressure. At our center, it was used in all our pediatric RAKT procedures. Bleeding can be particularly dangerous in children, as their already limited circulating volume causes any unrecognized or uncontrolled bleeding to be hazardous to cardiovascular function (32). Pediatric abdominal cavities typically contain adjacent organs in tight proximity to each other, increasing the risk for damage to surrounding organs during surgical procedures. Therefore, surgeons should be aware that open placement of the first trocar under direct vision (Hasson technique) is the preferred method for the pediatric population (33, 34).

Trocar positioning is also important for maximization of robot dexterity within the already restricted pediatric abdominal cavity. The recommended 8 cm gap for collision avoidance between the robot arms in adult RALS cases is often impossible in children due to workspace space limitations. Contrary to some adult procedures, there is no standardized trocar placement in robotic pediatric procedures due to broad variations in weight and height which are dependent on patient age. Trocar positioning should therefore be individually adjusted to allow optimal workspace (8). It is also worth to mention that the later generation robots (such as Da Vinci Xi and X) facilitate the docking procedure related to easy connection of instruments and the increased movement ability of the patient cart.

Additionally, while most robotic adult procedures involve four robot arms, the limited workspace in pediatric patients commonly only allows usage of three out of four robot arms (camera port and two robotic ports) without any additional 5-mm assisting port (8). In our initial phase of RAKT in children, our team opted for four robot arms with two assisting ports (the standardized adult port position) to limit operative time and more specifically to limit the (re)warm(ing) ischemia time, as these factors are potentially associated with adverse long-term patient and graft survival outcomes (35).

## Economic Considerations

Current studies have not found RALS to be cost-effective (6). It has been suggested that RALS may prove cost-effective in high-volume centers with at least 349 annual interventions (36). Therefore, cooperation between pediatric and adult urology units might be necessary as the volume for pediatric urology RALS is not as high as it is in adults (8). RALS may become more cost-effective as more experience is gained, leading to improved outcomes and reduced length of hospital stay. This issue remains to be further evaluated.

**Figure 1:**
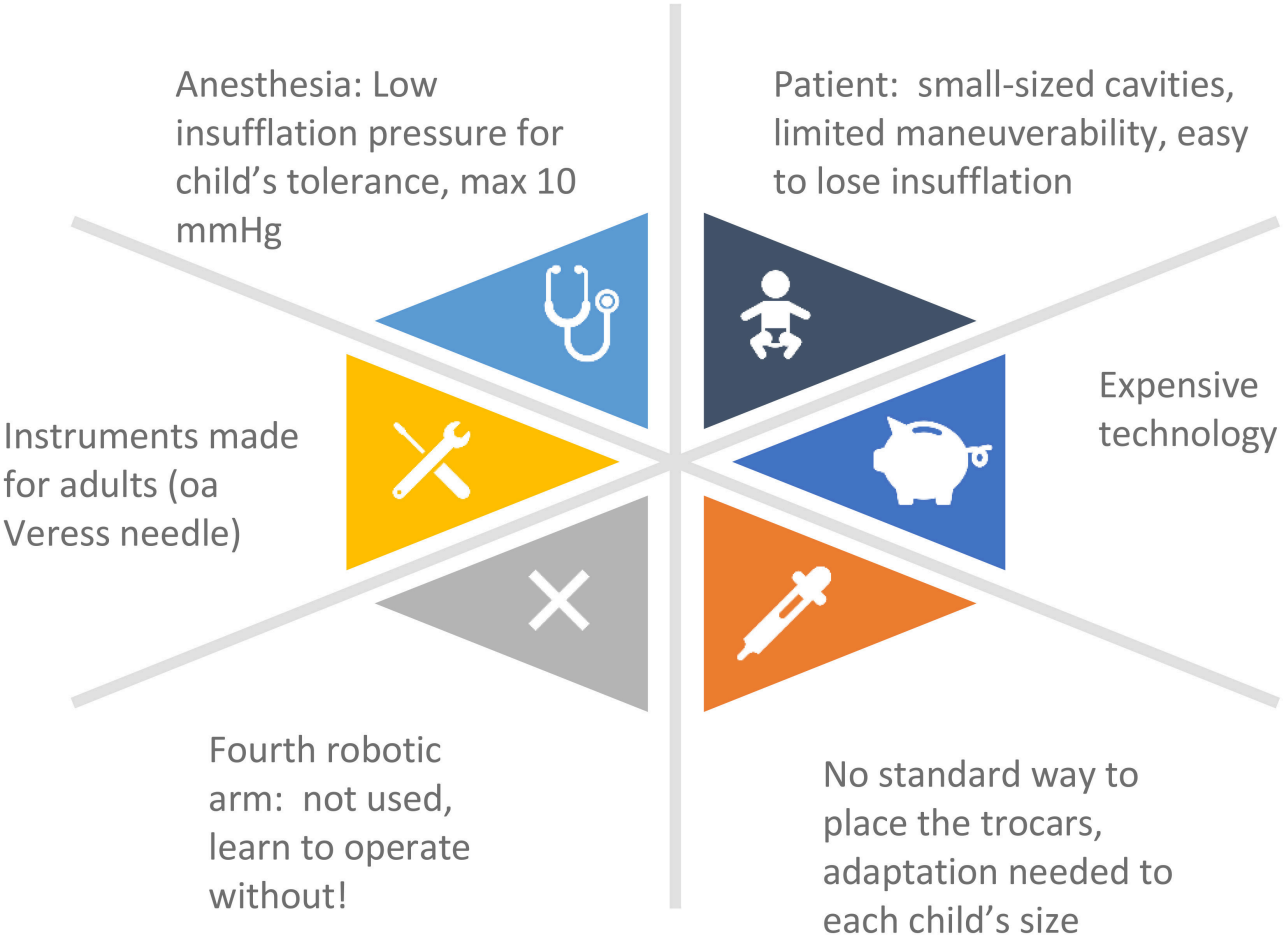
Challenges to be encountered when adapting the robotic platform to infants.

Figure 1, also published in a previous paper of our group (8), provides a diagrammatic overview of the main challenges in adapting the robotic platform to children.

## Outcomes

The differences in both the esthetic and functional outcomes of RAKT as compared to OKT must not be underestimated (21, 37, 38). RAKT can deliver superior esthetic outcomes, which are especially desired for young children (21). Barbosa et al. (38) demonstrated that parents consistently perceive smaller scars resulting from RALS as esthetically superior to open surgical scars following pediatric urological procedures. Regardless of scarring, most parents ultimately base their choice of surgical technique on superiority of clinical outcomes (38). Surgeons should therefore discuss differences in clinical outcomes with their patient's parents, in addition to the resultant scarring of different approach modalities.

To date, an eight kilograms cut-off value is the lowest weight allowed for consideration of robot-assisted minimally invasive surgery in children. For RAKT specifically, 8 years of age is considered to be the minimum threshold, as most transplanted kidneys are adult-sized kidneys which are then transplanted into a smaller child. At our center, we were able to adopt the standard trocar configuration from adult RAKT to pediatric RAKT up to a lower age limit of 8 years old. Extending to younger children would necessitate further adjustment of the trocar configuration, but would as well be very challenging or impossible due to the size of an adult-sized kidney in the particularly small pneumoperitoneum space of a small child. Therefore, 8 years of age is currently considered to be the minimum threshold for RAKT.

As the length of the incision in open surgery in smaller children is limited, there is discussion whether this population would benefit from this approach. While RA laparoscopic approach can provide better visualization with decreased inflammatory reaction and wound tension, opponents argue that incisions for open surgery are already small in size and that the sum of laparoscopic incisions equals the total length of an open incision (8, 39). However, this argument assumes that surgical morbidity is proportional to the sum of several incisions. However, morbidity has already proven to be a linear function of the tension present across the incision. As calculated by Blinman (37), an open incision is consistently subject to more total closing tension than any combination of trocar incisions of equal total length. By decreasing wound tension, there is an important reduction in pain and morbidity (risk of dehiscence, hernia or infection) (37).

## Training and Experience

In order to start a pediatric RAKT program, a team of surgeons experienced both in RALS and kidney transplantation surgery, along with the full support of a pediatric nephrology and anesthesiology team, is recommended. Even during the training phase of learning how to perform RAKT, the safety of the procedure and optimal graft survival should be guaranteed.

According to Decaestecker et al. (27), it cannot be emphasized enough that a high level of robotic experience is recommended before initiating a RAKT program. Training the technique on both dry and wet lab models is mandatory (27). The only structured RAKT course, provided at ORSI Academy (Melle, Belgium) (40), is recommended. And additional structured RA pediatric course is available at the same place, and is highly recommended. In conjunction with an experienced RAKT proctor supporting first cases, a safe introduction of this new technique is possible (16, 27, 41, 42).

## Specifics Requiring Special Attention in Pediatric RAKT

Warm ischemia time has already been shown to be a negative predictive factor of long-term patient and graft survival outcomes (35). In the adult RAKT series, a wide range of ischemia times has been described, but no correlation has been found between rewarming time and graft function (16, 17). To reduce potential damage after prolonged warm ischemia times, Menon et al. have reported a new technique for regional hypothermia with ice slush during vascular anastomosis (15).

Contrary to the adult RAKT procedure, in pediatric RAKT the graft renal vessels are anastomosed to the common iliac vessels instead of the external iliac vessels, in order to match the vessel size of the donor (adult-sized renal vessels) and acceptor (pediatric-sized iliac vessels) (20, 21).

It should be noted that according to the literature, renal blood flow and function are reduced in the presence of pneumoperitoneum, potentially leading to graft impairment (43). The potential damage to the graft as a result of pneumoperitoneum is not yet fully known, and a correlation between operative time (and thus exposure to pneumoperitoneum) and graft function in adult RAKT has not yet been identified (16). Aside from the anesthetic concerns regarding intra-abdominal pressure, this might be another reason to keep intra-abdominal pressure around 10 mmHg or lower. We prefer a stable low pneumoperitoneum pressure of 8 mmHg for the (RA) laparoscopic living donor nephrectomy and for RAKT as of the moment of reperfusion to mitigate any potential adverse influence of higher pneumoperitoneum pressures in donor and/or recipient (44, 45).

## Complications and Length of Hospital Stay

Differences regarding complications and length of hospital stay after RAKT as compared to OKT are still subject to debate. To date, RAKT in children remains in the initiation phase. A larger patient database could lead to more valuable conclusions. A randomized controlled trial comparing outcomes of RAKT and OKT should provide the best level of evidence.

## Conclusion

Although data on RAKT in children is scarce, it is a safe and feasible procedure and results in excellent graft function. It should only be performed by a RAKT team experienced in both robot-assisted laparoscopic surgery and transplantation surgery, fully supported by a team of pediatric nephrologists and anesthesiologists. Further research is necessary to better determine the value of the robotic approach as compared to the laparoscopic and open approach. Cost-effectiveness will remain an important subject of debate and is in need of further evaluation as well.

## Data Availability Statement

The original contributions generated for this study are included in the article/supplementary material, further inquiries can be directed to the corresponding author/s.

## Author Contributions

JG was the main author and carried out literature search. KD and A-FS performed the initial surgery reported in Spinoit et al. (20). A-FS initiated the scientific project and develops the pediatric robotic program of minimally invasive surgery. KD initiated the scientific and develops the surgical robotic kidney transplantation program in Ghent. AB and MS are promotors of the international robotic programs, respectively for Kidney transplant and for pediatric urology. They critically revised the manuscript and approved the submitted version. All authors from Ghent are part of the pediatric uro-nephrology team making the development of the program possible and critically revised the manuscript and approved the submitted version.

## Conflict of Interest

KD is a consultant for Intuitive Surgical, Sunnyvale, CA, United States of America. The remaining authors declare that the research was conducted in the absence of any commercial or financial relationships that could be construed as a potential conflict of interest.
